# Supplementary material for: Wnt signaling restores evolutionary loss of robust foot regeneration rates in Hydra
Source: Nat Commun. 2025 Dec 10;16:11447. doi: 10.1038/s41467-025-66299-2 (PMC12748876; doi:10.1038/s41467-025-66299-2)
Supplement: Supplementary file 1 — Supplementary Information [file 41467_2025_66299_MOESM1_ESM.pdf]

Wnt signaling restores evolutionary loss of robust foot regeneration rates in *Hydra*

Sergio E. Campos, Sahar Naziri, Jackson Crane, Jennifer Tsverov, Ben D. Cox, Craig Ciampa, Ruthie B. Spencer, Robert E. Steele, Daniel E. Martínez & Celina E. Juliano

Supplementary Information

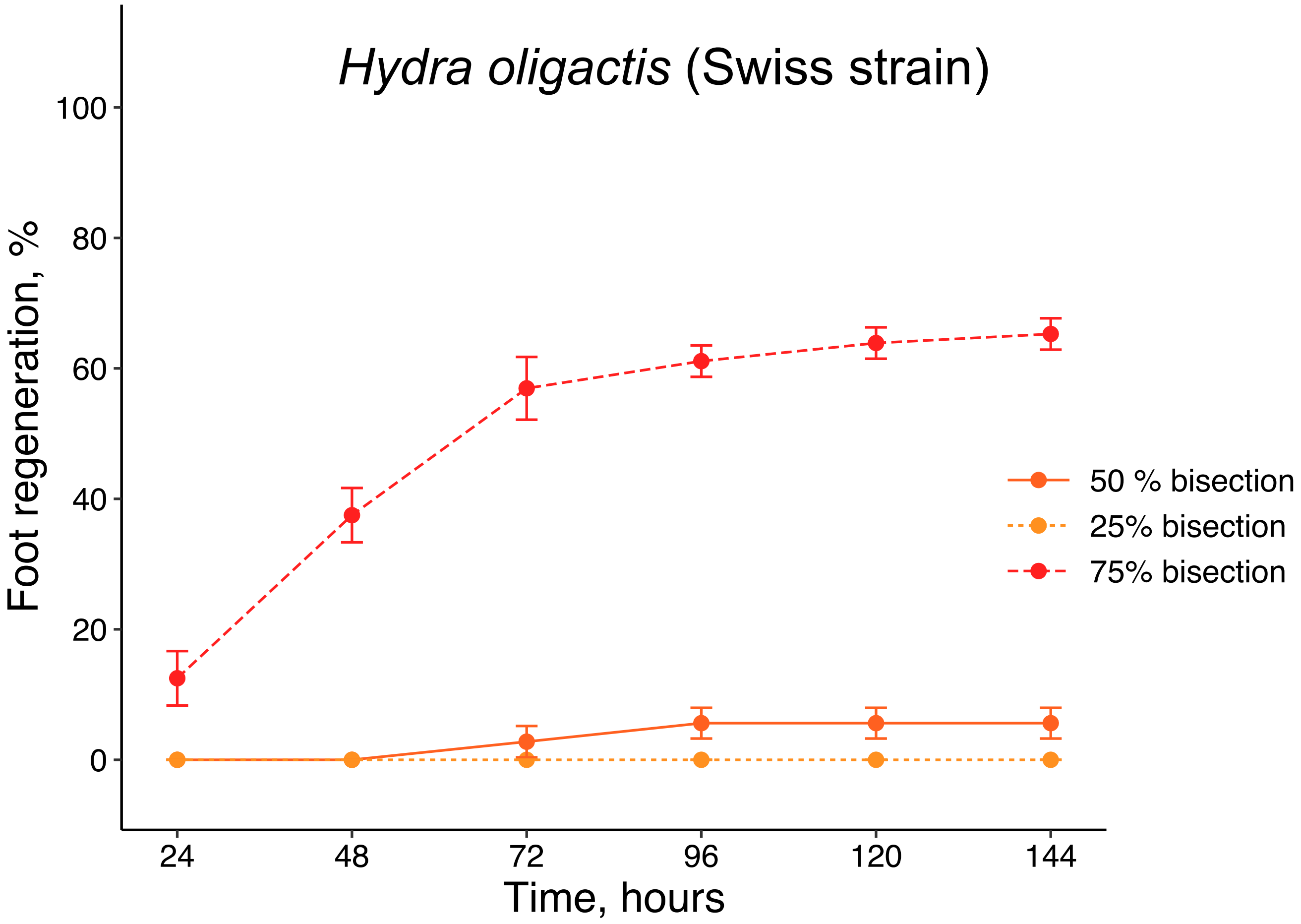

Supplementary Fig. 1. The *Hydra oligactis* Swiss strain displays low foot regeneration rates similar to the Innsbruck strain. Line plots showing mean foot regeneration percentage in *H. oligactis* Swiss strain with bisections at different body lengths. Approximately 5% of *H. oligactis* Swiss strain polyps bisected at 50% body length successfully regenerated their feet (solid orange line), while the remaining ~95% remained footless. In polyps bisected at 25% body length, 100% remained footless (light orange dotted line). Polyps bisected at 75% body length exhibited a higher foot regeneration potential, with over 60% successfully regenerating feet (dark orange dashed line). See Figure 1F for diagram of amputation sites. Three biological replicates were performed for each type of bisection (50% bisection: n_1 = 24, n_2 = 24, n_3 = 23. 25% bisection: n_1 = 24, n_2 = 24, n_3 = 24. 75% bisection: n_1 = 24, n_2 = 24, n_3 = 24). Error bars represent standard deviation of the three replicates and the data points represent the average percentage of foot regeneration at each time point. The underlying data for this figure is provided in Source Data File 2.

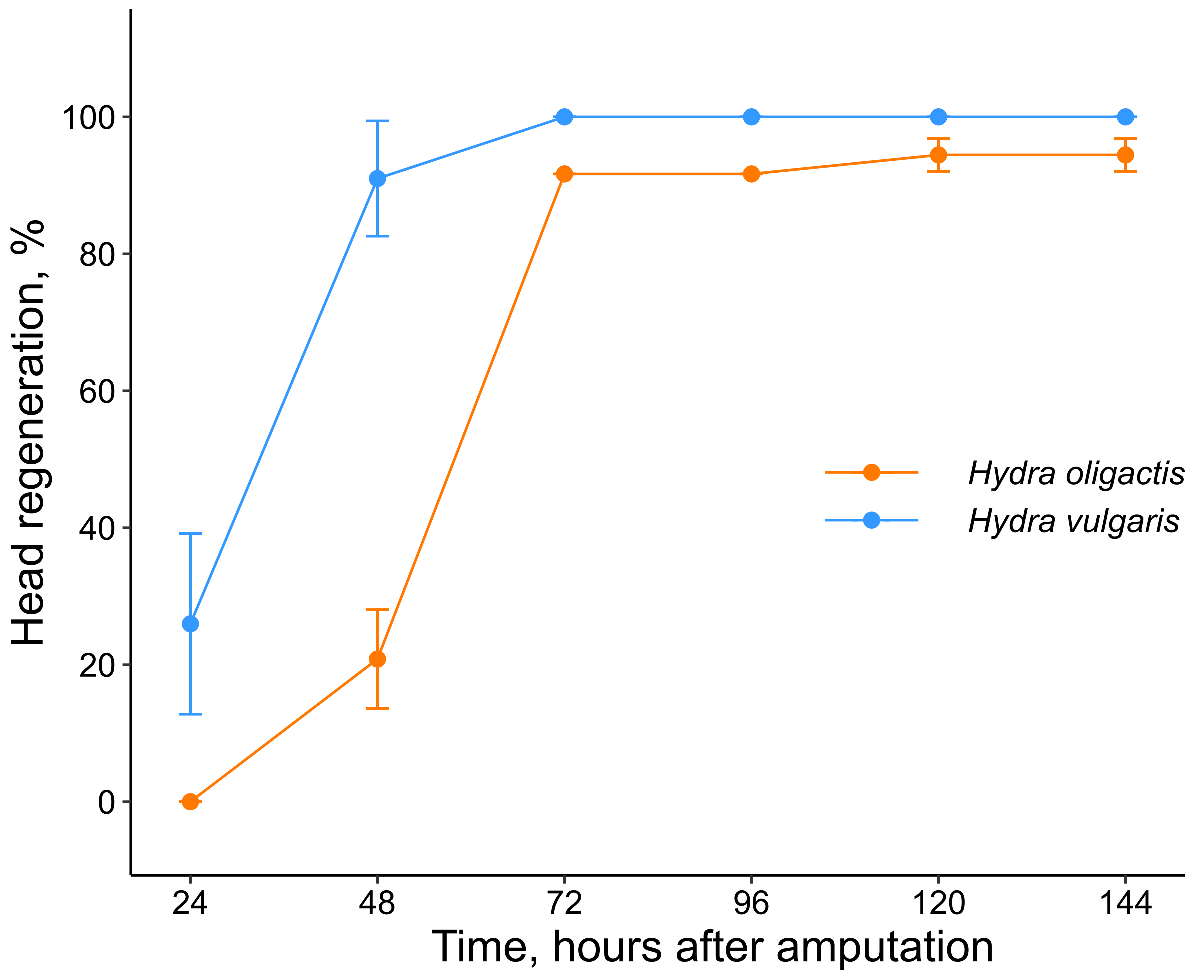

Supplementary Fig. 2. Head regeneration occurs at a slower rate in *H. oligactis* Innsbruck 12 strain as compared to *H. vulgaris*. Line plots showing mean head regeneration in *H. oligactis* and *H. vulgaris*. By 48 hpa, approximately 90% of bisected *H. vulgaris* regenerate their heads as determined by the appearance of tentacle buds (blue line), whereas only about 20% of *H. oligactis* achieve head regeneration within the same time frame (orange line). Three biological replicates were performed for each species (*H. oligactis*: n_1 = 24, n_2 = 24 and n_3 = 24. *H. vulgaris*: n_1 = 29, n_2 = 29 and n_3 = 29). Error bars represent the standard deviation of the mean of three biological replicates and the data points represent the average percentage of head regeneration at each time point. The underlying data for this figure is provided in Source Data File 2.

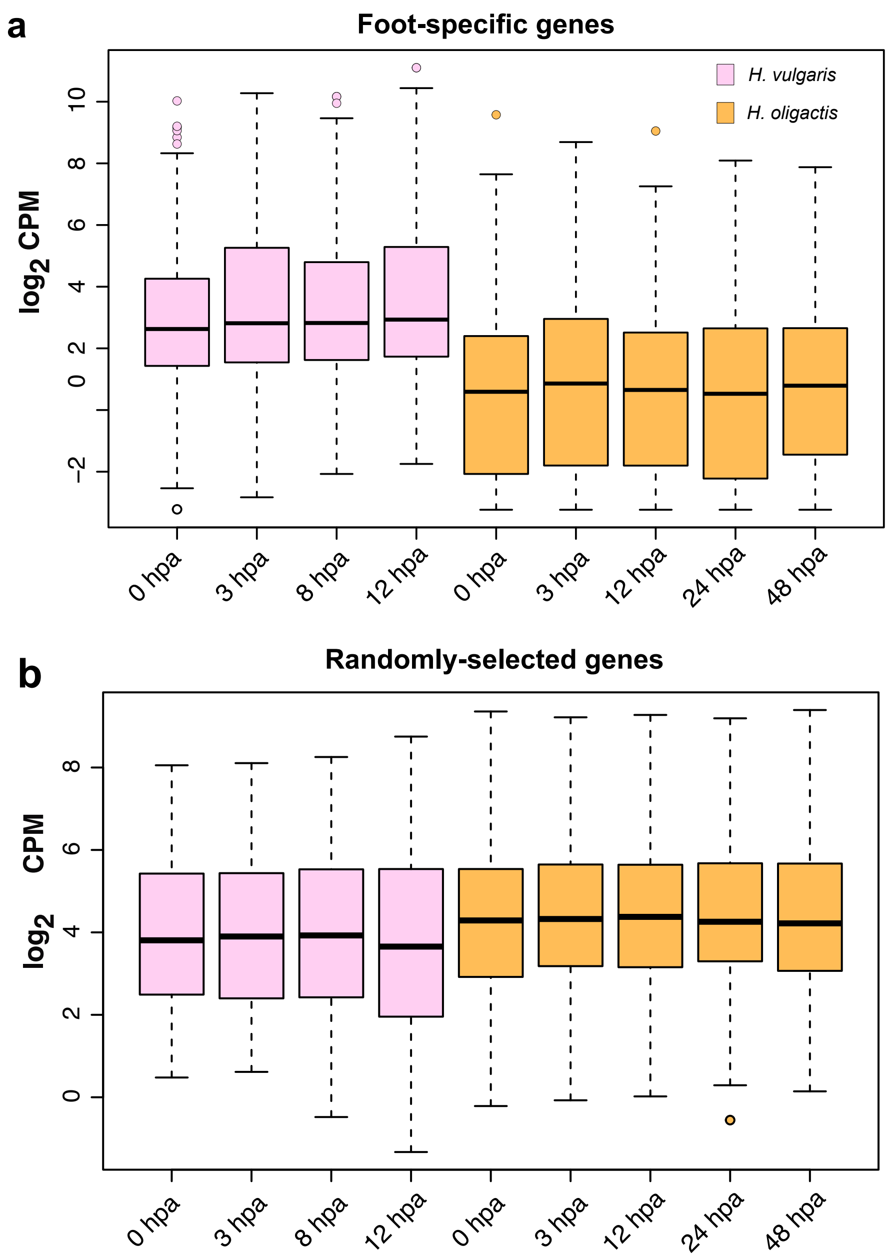

Supplementary Fig. 3. Foot regeneration genes are expressed at lower levels in *H. oligactis* compared to *H. vulgaris*. (**a**) Boxplots showing expression levels of 154 orthologs from the foot-specific gene list identified between *H. oligactis* and *H. vulgaris* reference transcriptomes. (**b**) Boxplots of 63 randomly selected orthologs between *H. oligactis* and *H. vulgaris* plotted as controls. The log2 counts per million (CPM) for these transcripts were plotted over the course of foot regeneration in *H. vulgaris* (pink boxplots) and *H. oligactis* (orange boxplots). Foot-specific genes were consistently expressed at lower levels in *H. oligactis* as compared to *H. vulgaris*, including at 0hpa. The boxes represent quartile group 2 and quartile group 3, the bold line represents the median log2 CPM, the whiskers represent the theoretical maximum and minimum values for the log2 CPM distribution. Outliers are shown beyond the whiskers. Randomly sorted genes were taken from the foot- specific genes worksheet in Supplementary Data file 1. The data for the genes plotted in this figure is provided in Source Data file 17.

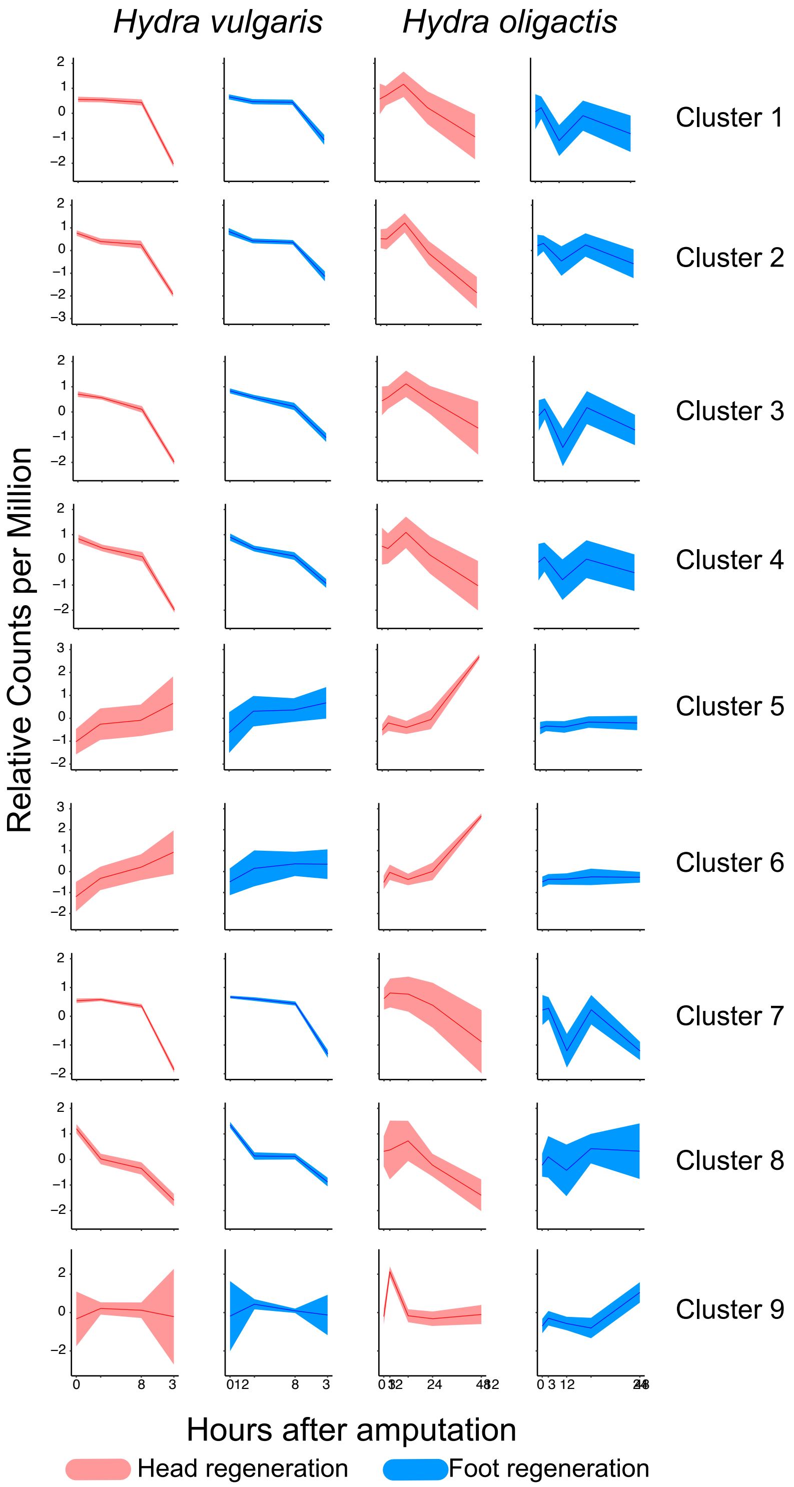

Supplementary Fig. 4. Gene co-expression analysis using the OrthoClust pipeline identified nine distinct clusters of orthologous genes with varying expression patterns between *H. oligactis* and *H. vulgaris*. Red ribbon plots depict the mean expression patterns of clustered genes in oral regenerating tissue, while blue ribbon plots represent expression patterns in aboral injured tissue. Expression profiles for *H. vulgaris* clusters are shown on the left, corresponding profiles for *H. oligactis* are shown on the right. Note that the *H. oligactis* RNA-seq data spans a longer time frame (0-48 hpa) as compared to the *H. vulgaris* data (0-12 hpa). Light colored shading represents standard deviation. The underlying gene expression data used to conduct this analysis is provided in Supplementary Data File 5.

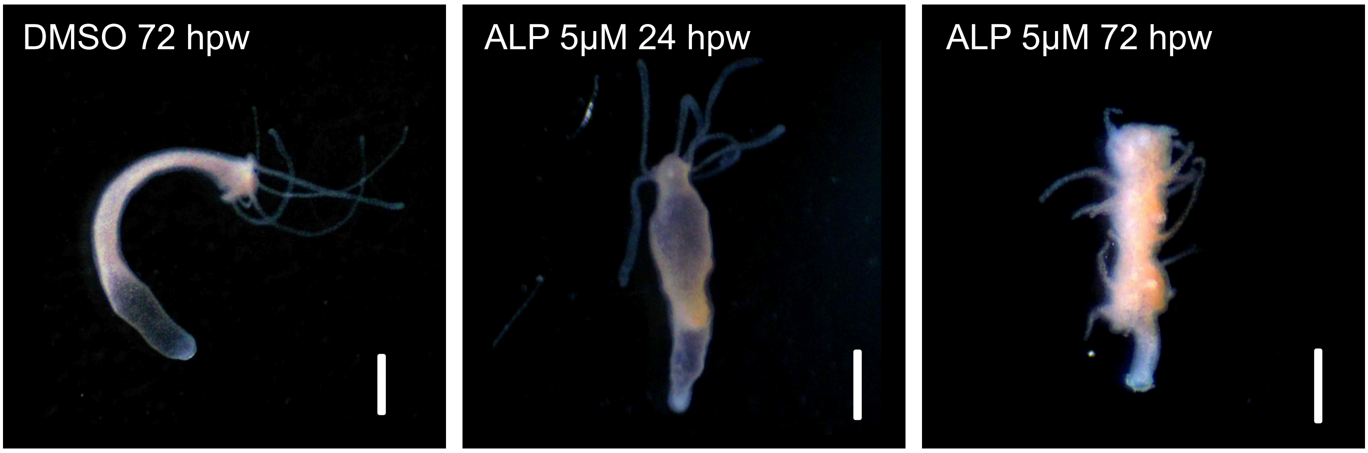

Supplementary Fig. 5. Wnt activation with Alsterpaullone (ALP) induces ectopic tentacle formation in uninjured *H. oligactis.* Thirty *H. oligactis* polyps were treated with either DMSO or 5 μM ALP for 24 hours, followed by washing into *Hydra* medium. The left panel shows the typical phenotype of DMSO-treated animals 72 hours post-wash (hpw). The middle panel shows the phenotype observed in 26 of 30 animals at 24 hpw. The right panel shows the ectopic tentacle formation, present in 22 out of 30 animals at 72 hpw. Scale bar = 1 mm.

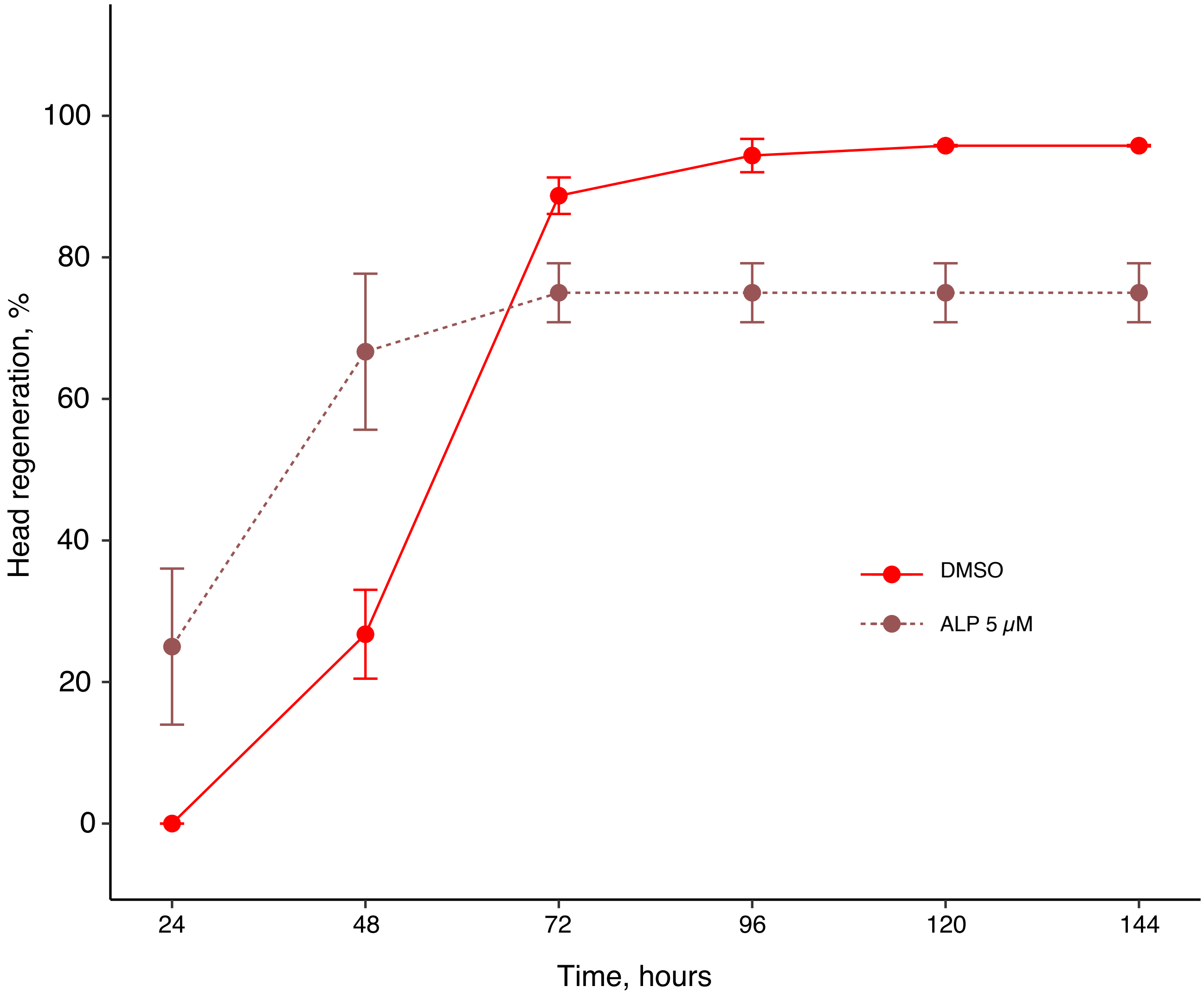

Supplementary Fig. 6. Wnt activation accelerates head regeneration in *H. oligactis*. Line plots showing mean head regeneration in 5 μM ALP-treated bisected *H. oligactis* and DMSO-treated controls. Head regeneration was scored by the presence of tentacle buds. In DMSO-treated controls, no animals showed tentacle buds at 24 hpa, and only ~20% showed tentacle buds by 48 hpa (solid red line). In contrast, treatment with 5 μM ALP resulted in ~20% of animals showing tentacle buds at 24 hpa and over 60% by 48 hpa (dotted brown line). Three biological replicates were conducted for each condition (DMSO: n_1 = 23, n_2 = 24 and n_3 = 24. ALP 5μM: n_1 = 24, n_2 = 24 and n_3 = 24). The error bars represent the standard deviation of the three replicates and the data points represent the average percentage of head regeneration at each time point. The underlying data for this figure is provided in Source Data file 18.

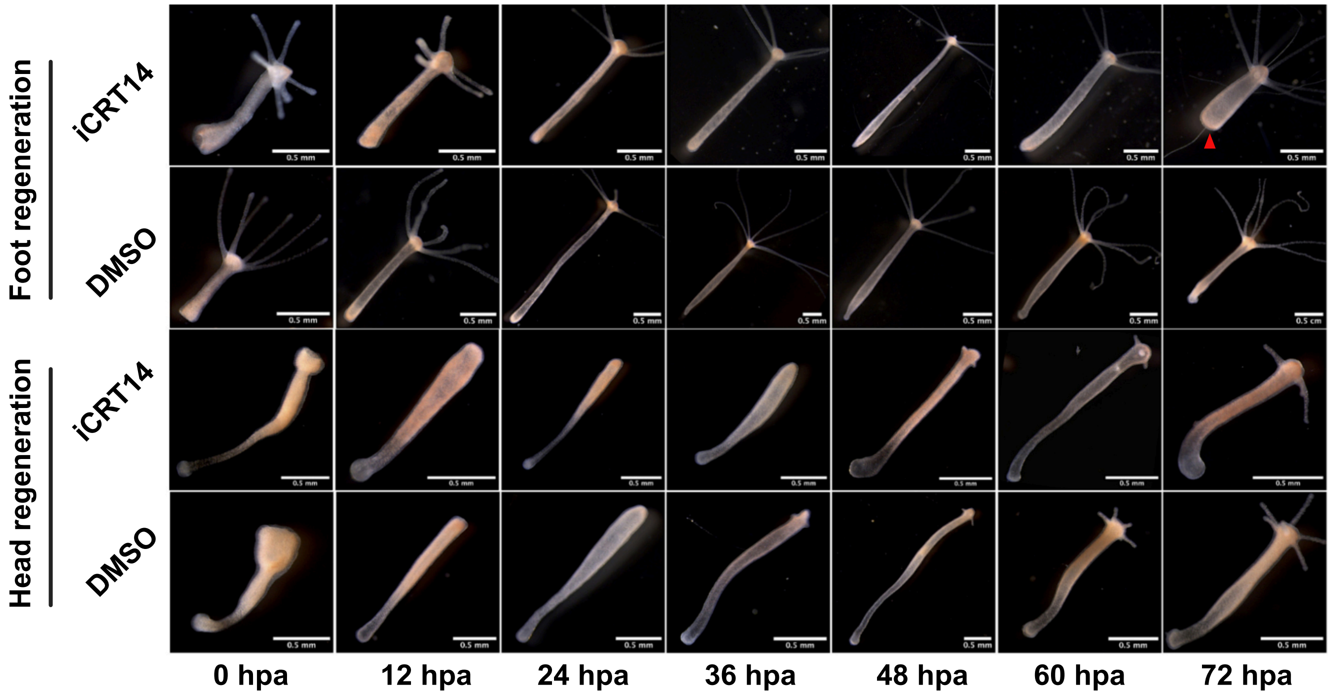

Supplementary Fig. 7. Wnt inhibition blocks foot regeneration and delays head regeneration in *H. vulgaris*. *H. vulgaris* polyps were pre-treated with 5 μM iCRT14 for 2 hours prior to 50% bisection and maintained in iCRT14 for 12 hours after injury. Representative images illustrate foot regeneration (top) and head regeneration (bottom) over the time course. Foot regeneration is inhibited by iCRT14 compared to DMSO treated animals (See Figure 4b), with inhibited animals becoming stably footless (red triangle points at a footless aboral end in treated *Hydra* at 72 hpa). Head regeneration showed a delay in the first appearance of tentacles in iCRT14-treated samples as compared to DMSO treated animals (See Figure 4c). The animals photographed are representative images of the the experiment detailed in the main text for Figure 4b and 4c. In brief, the experiment was performed with three biological replicates with n=89 for iCRT14-treated animals and n=87 for DMSO-treated animals.

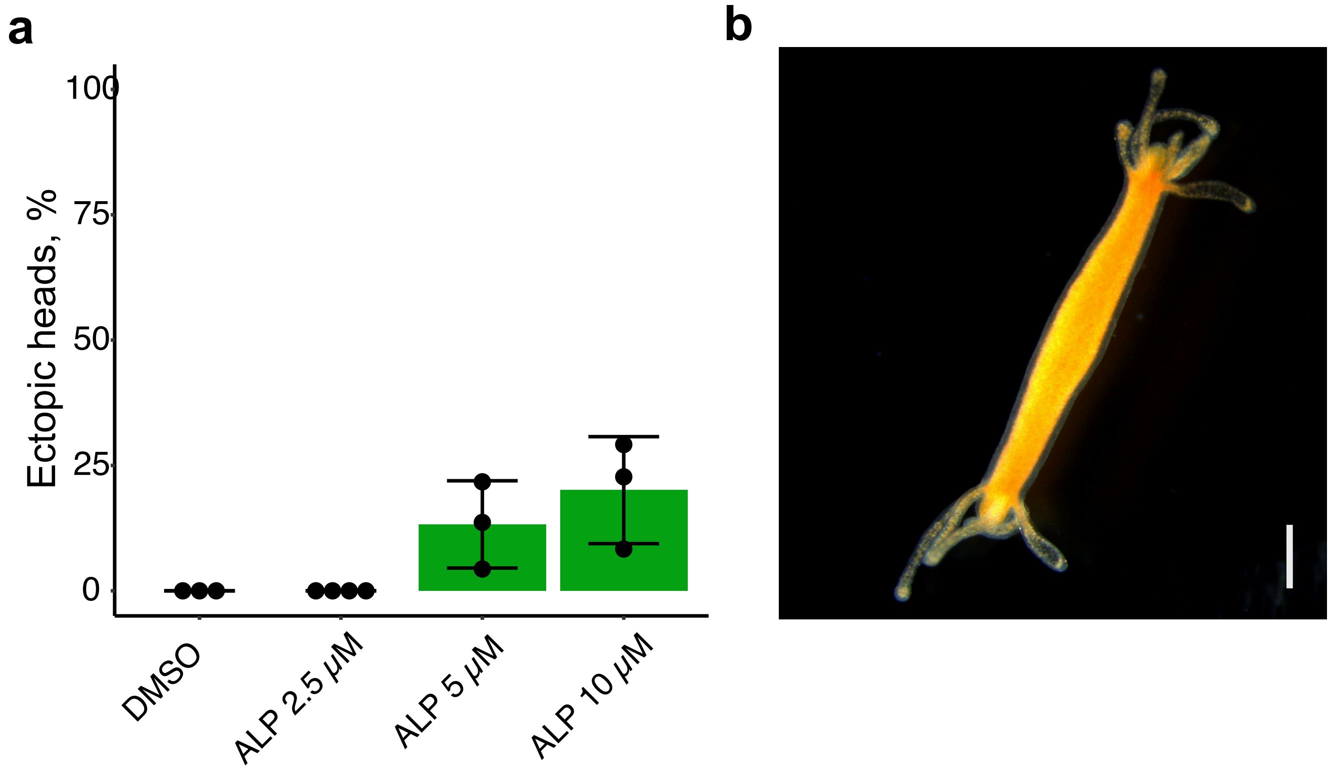

Supplementary Fig. 8. High Wnt signaling activation induces ectopic head regeneration at the aboral wound site. a Bar plot showing the mean percentage of ectopic head regeneration in *H. oligactis* upper halves treated for 3 hours with different concentration of ALP. Foot regeneration (see Fig. 4) and ectopic head formation at the aboral wound site was assessed 6 days after injury. At least three biological replicates were used to calculate the foot regeneration percentage in *H. oligactis* for each treatment: DMSO (n_1 = 26, n_2 = 24 and n_3 = 24), ALP 2.5μM (n_1 = 21, n_2 = 21, n_3 =23 and n_4 = 24), ALP 5μM (n_1 = 23, n_2 = 23 and n_3 = 22) and ALP 10μM (n_1 = 24, n_2 = 22 and n_3 =24). The error bars represent the standard deviation of the mean ectopic head percentage values for the different biological replicates. The underlying data for this plot is provided in Source Data file 12. **b** Representative image of an ectopic head regenerated at the aboral wound site in *H. oligactis* treated with 10 μM ALP. The image was taken 14 days post-injury. Scale bar: 1 mm.

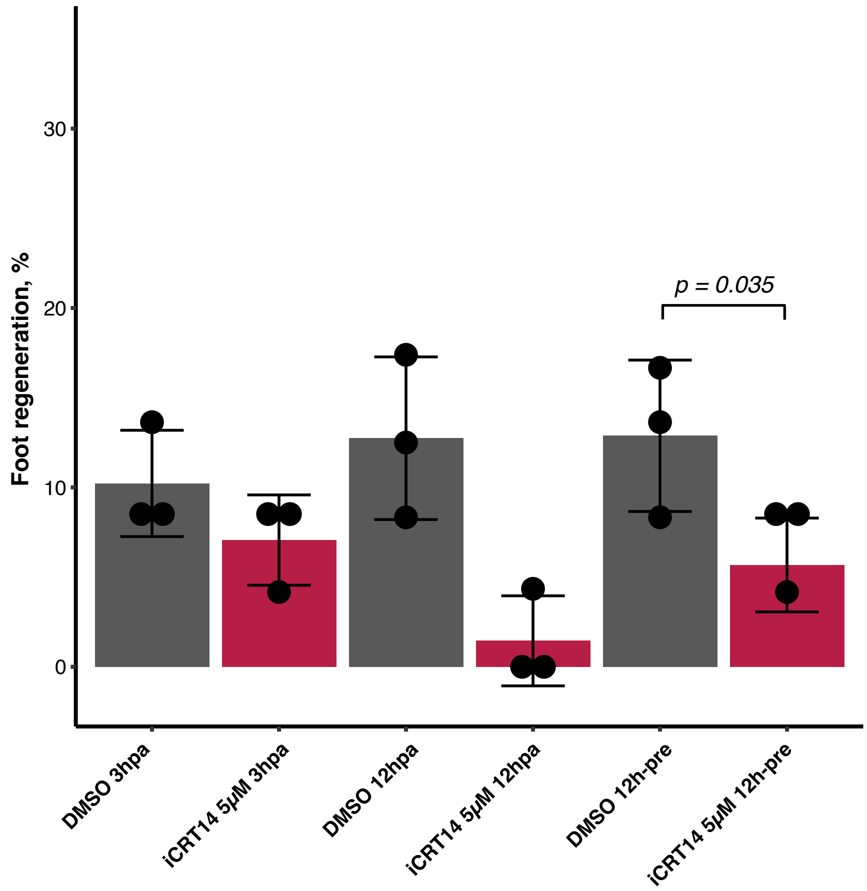

Supplementary Fig. 9. Wnt signaling inhibition with iCRT14 modestly reduces foot regeneration rates in *H. oligactis*. Bar plot showing the mean percentage of regenerated feet after 50% bisection in *H. oligactis* upper halves treated with either 5μM iCRT14 or 0.05% DMSO. The details for the treatment schedule were as follows: iCRT14-treated animals incubated in the drug for 3 hpa (with a 2-hour incubation prior to amputation) were tested with three biological replicates (n_1 = 24, n_2 = 23 and n_3 = 24); DMSO-treated control animals incubated for 3 hpa (with a 2-hour pre-incubation) were tested with three biological replicates (n_1 = 22, n_2 = 24 and n_3 = 23); iCRT14-treated animals incubated in the drug for 12hpa (with a 2-hour pre-incubation) were tested with three biological replicates (n_1 = 24, n_2 = 23 and n_3 =22); DMSO-treated control animals incubated for 12 hpa (with a 2-hour pre-incubation) were tested with three biological replicates (n_1 = 24, n_2 = 24 and n_3 = 23); iCRT14-treated animals incubated in the drug for 12 hours prior to amputation (no pre-incubation) were tested with three biological replicates (n_1 = 24, n_2 = 24 and n_3 = 23); finally, DMSO-treated control animals incubated for 12 hours prior to amputation (no pre-incubation) were tested with three biological replicates (n_1 = 22, n_2 = 24 and n_3 = 24). Grey bars represent DMSO and red bars represent iCRT14-treated samples. Error bars represent the standard deviation of the mean foot regeneration percentage of each replicate for each condition. Foot regeneration rates were only significantly different between control and iCRT14 treatment for 12 hours with a 2-hour pre-incubation time according to a t-test (p-value= 0.03562, t=5.1562, df=2, 95 percent confidence interval: 0.9623389 10.6648021, mean difference=5.81357). The underlying data for this figure are provided in Source Data File 19.

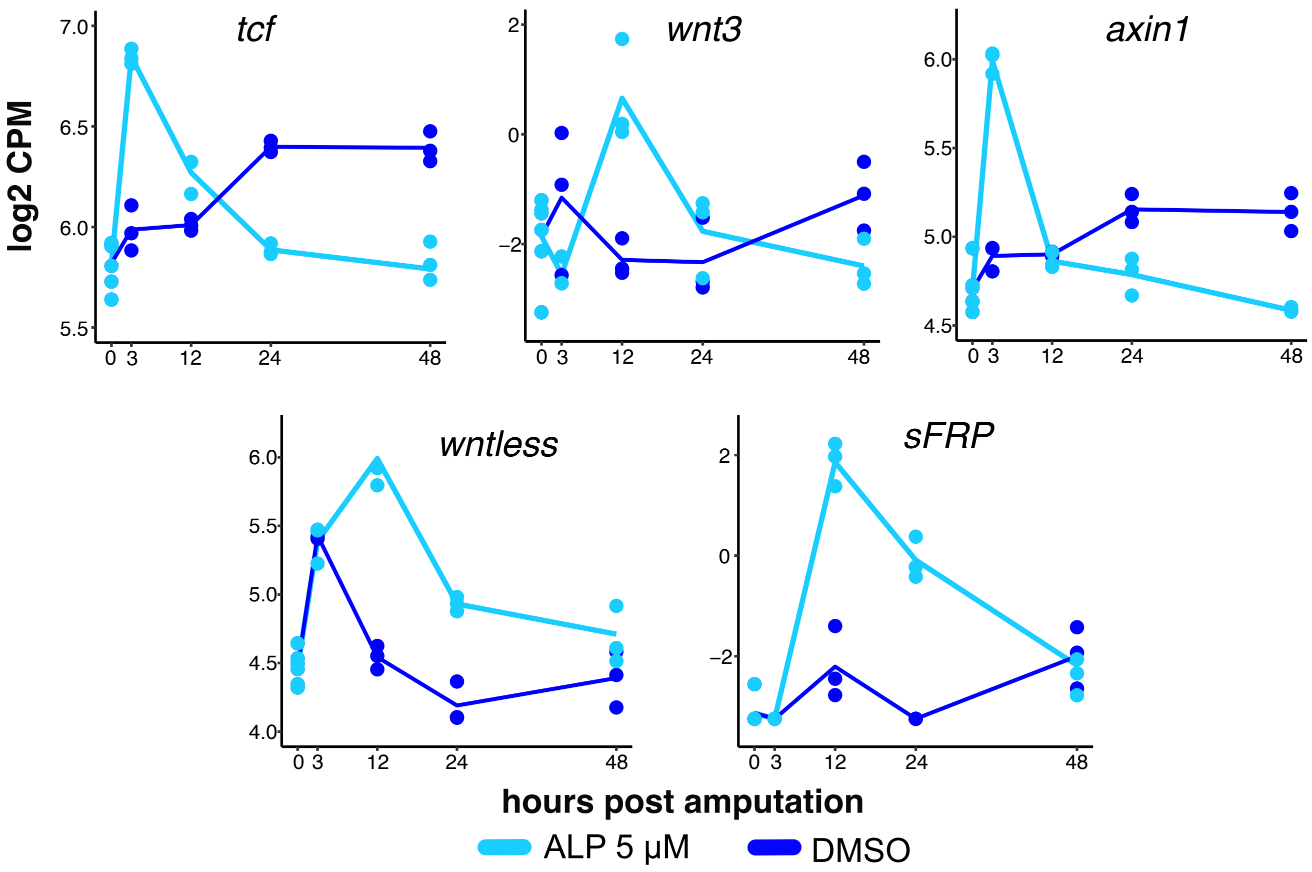

Supplementary Fig. 10. ALP treatment induces the transcriptional activation of Wnt pathway genes. RNA expression plots show the mean $\log_2$ counts per million ($\log_2$ CPM), from three biological replicates (n=30 animals each replicate), for Wnt pathway genes *tcf, wnt3, axin1, wntless* and *sFRP*. The blue line represents expression in DMSO-treated failed foot regenerating tissue; the light blue line represents expression in ALP-treated foot regenerating tissue. Log2CPM values for these plots are provided in Source Data File 13.

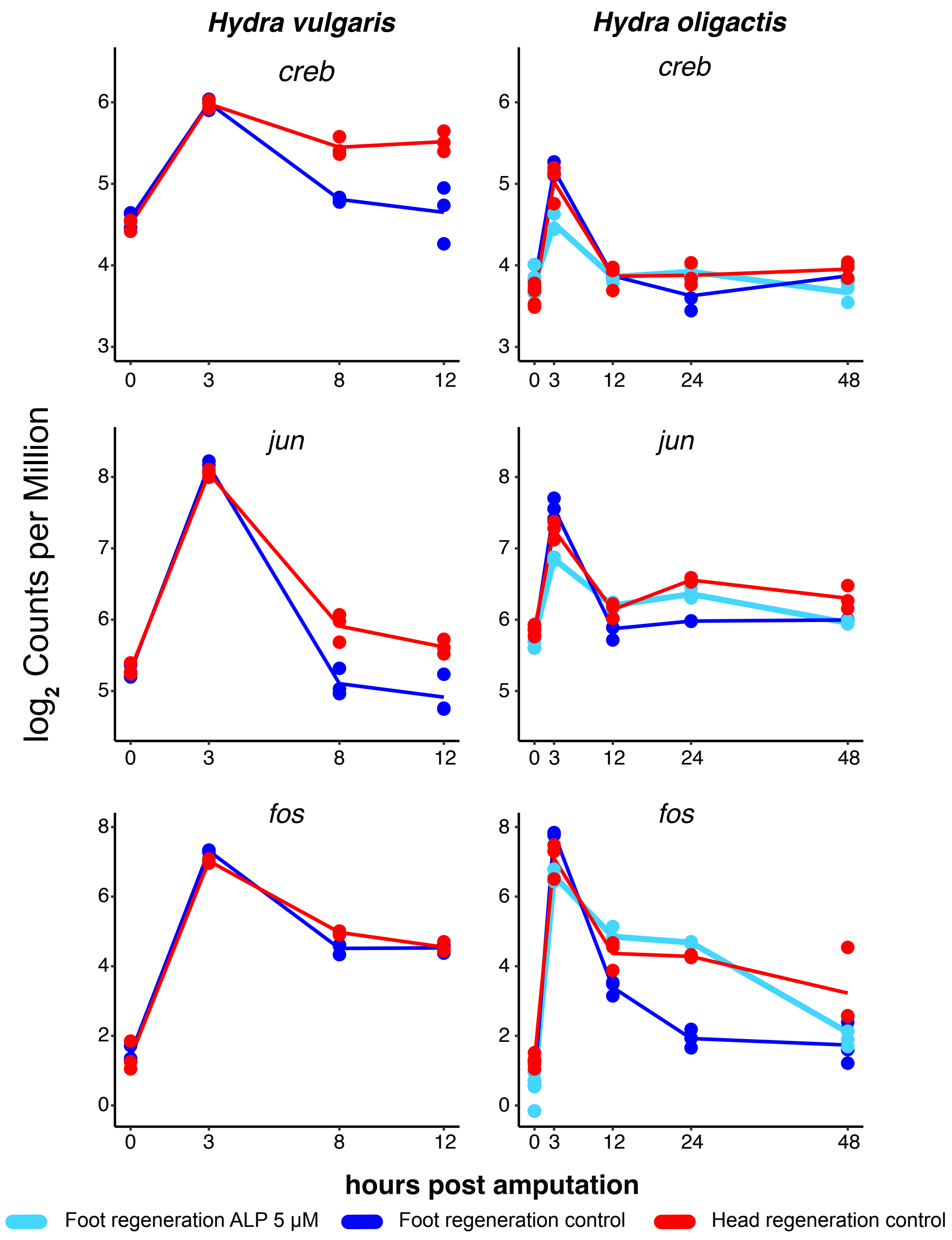

Supplementary Fig. 11. bZIP transcription factors are downregulated with ALP treatment at 3hpa. RNA expression plots show the mean $\log_2$ counts per million ($\log_2$ CPM), from three biological replicates (n=30 animals each replicate) for bZIP transcription factor genes *creb, jun* and *fos* in *H. vulgaris* (left) and *H. oligactis* (right). For *H. vulgaris* the blue line represents gene expression in foot regenerating tissue and the red line represents gene expression in head regenerating tissue. For *H. oligactis*, the blue line represents gene expression in DMSO-treated failed foot regenerating tissue, the light blue line represents gene expression in ALP-treated foot regenerating tissue, and the red line represents gene expression in DMSO-treated head regenerating tissue. *H. oligactis* log2 CPM values for these plots are provided in Source Data File 4 and Source Data File 13. *H. vulgaris* log2 CPM values for these plots are provided in Source Data File 9.

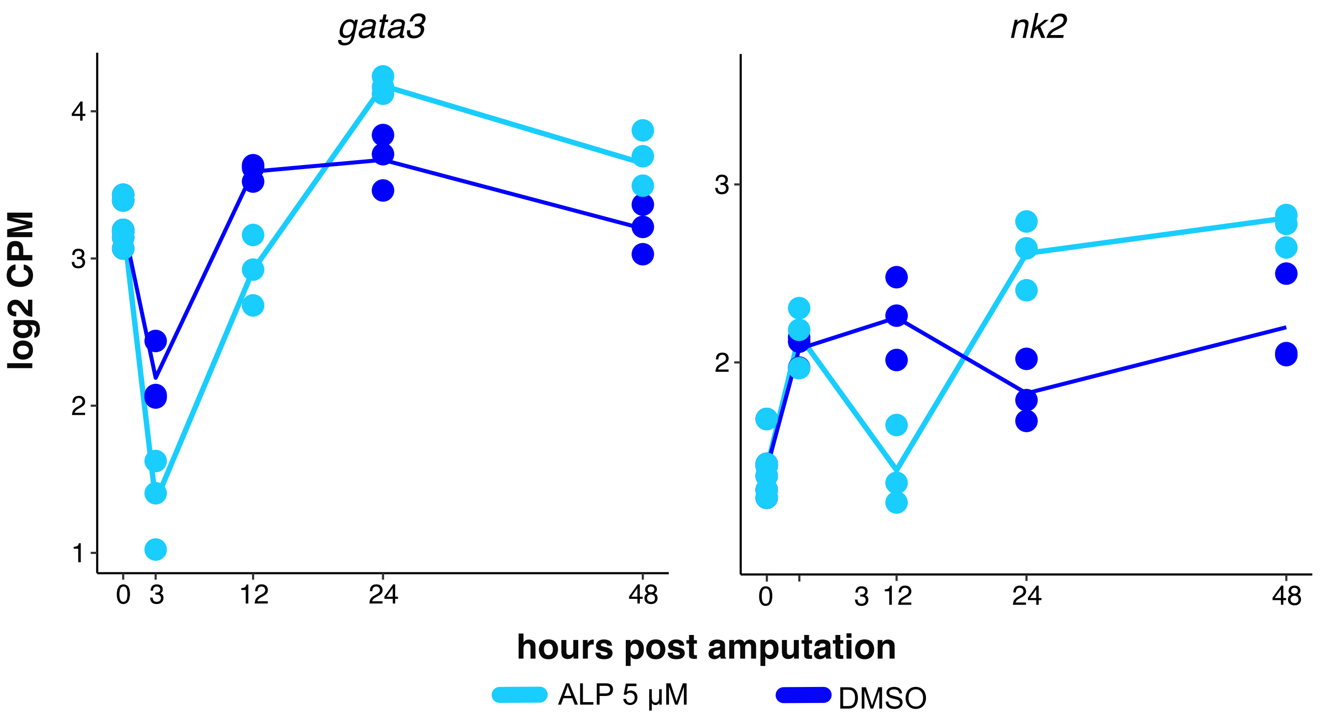

Supplementary Fig. 12. ALP promotes the upregulation of foot-specific TFs at 24 hours post amputation. RNA expression plots show the mean $\log_2$ counts per million ($\log_2$ CPM) for foot-specific transcription factors genes *gata3* and *nk2*. The blue line represents expression in DMSO-treated failed foot regenerating tissue; the light blue line represents expression in ALP-treated foot regenerating tissue. Log2 CPM values for these plots are provided in Source Data File 13.

Supplementary Table 1. Comprehensive list of standard genes discussed in the manuscript. The gene identities for *H. oligactis* transcripts were obtained by reciprocal blast against gene models in *H. vulgaris* and orthogroups obtained from an OrthoFinder analysis that included 44 animal transcriptomes[1]. The transcriptomic reference for *H. oligactis* used in this study is provided in Supplementary data File 8.

Gene name	Transcript ID (*H. oligactis)*	Gene model ID (*H. vulgaris)*
ywhaz	R035432c1g1_i02	g14670
aatf	R038486c1g1_i01	g22584
axin1	R031348c0g1_i01	g14938
brachyury1	R035976c0g2_i01	g24952
creb	R027355c0g1_i03	g16491
dmrta2	R023528c0g1_i01	g3847
fd4	R032408c0g1_i01	g28449
fos	R029832c0g1_i01	g23720
foxl1	R005786c0g1_i02	g32304
gata3	R023611c0g1_i01	g20911
dll1	R017255c0g1_i02	g6880
dlx1	R035295c0g1_i01	g23296
dlx2	R029042c0g1_i02	g18245
jun	R013156c0g1_i01	g2328
nkx2	R037127c0g1_i01	g31954
npyr4	R014795c0g1_i01	g18779
kiaa1958	R023095c0g1_i01	g2198
opn4a	R028454c0g1_i03	g13698
rfx1	R039495c1g1_i04	g17406
sFRP	R035800c0g3_i01	g21027
tcf	R035844c0g2_i01	g27364
tld	R035649c0g1_i01	g4917
tle3	R040370c0g1_i01	g25647
wnt3	R033453c0g1_i01	g28064
wnt7	R026000c0g1_i01	g29750
wnt9/10c	R035762c0g1_i01	g33373
wntless	R038745c0g1_i02	g18842

Supplementary Table 2. Transcriptomic reference completeness and redundancy was assessed using BUSCO. Statistics from BUSCO software before and after using Evidential Gene (v2017.12.21), showing the completeness (C) of the *H. oligactis* transcriptomic reference, number of single genes in BUSCO (S), duplicated genes (D), fragmented genes (F), missing genes (M) for a total n of 954 genes from metazoans.

Transcriptomic reference redundancy before EvidentialGene pipeline	
# of genes	C:93.7%[S:67.4%,D:26.3%],F:2.4%,M:3.9%,n:954
894	Complete BUSCOs ©
643	Complete and single-copy BUSCOs (S)
251	Complete and duplicated BUSCOs (D)
23	Fragmented BUSCOs (F)
37	Missing BUSCOs (M)
954	Total BUSCO groups searched

Transcriptomic reference redundancy after EvidentialGene	
# of genes	C:93.3%[S:90.9%,D:2.4%],F:2.4%,M:4.3%,n:954
890	Complete BUSCOs (C)
867	Complete and single-copy BUSCOs (S)
23	Complete and duplicated BUSCOs (D)
23	Fragmented BUSCOs (F)
41	Missing BUSCOs (M)
954	Total BUSCO groups searched

Results from dataset metazoa_odb10
