# Supplementary material for: Wnt signaling restores evolutionary loss of robust foot regeneration rates in Hydra
Source: Nat Commun. 2025 Dec 10;16:11447. doi: 10.1038/s41467-025-66299-2 (PMC12748876; doi:10.1038/s41467-025-66299-2)
Supplement: Supplementary file 3 — Description of Additional Supplementary Files [file 41467_2025_66299_MOESM3_ESM.pdf]

Desscription of the files contained in this folder:

Supplementary Data 1. Excel workbook containing two lists for foot-specific genes (Foot-specific genes sheet) and head-specific genes (Head-specific genes sheet) in H. oligactis. Cells with NA values in sheet columns represent Not Available values.

Supplementary Data 2. Comma separated values file containing genes loaded onto PC1 from a Principal Component Analysis from Head regeneration RNA-seq samples in H. oligactis. Cells with NA values in sheet columns represent Not Available values.

Supplementary Data 3. Comma separated values file containing genes loaded onto PC1 from Principal Component Analysis from Foot regeneration RNA-seq samples in H. oligactis. Cells with NA values in sheet columns represent Not Available values.

Supplementary Data 4. Excel workbook containing lists of Differentially Expressed Genes along with their functional annotations from all comparisons listed in the study. Each comparison is contained in a separate sheet: FR-specific_3hpa, contains genes up regulated specifically in foot regenerating tissue compared to head regenerating tissue at 3 hours post amputation (hpa). FR-specific_12hpa, contains genes up regulated specifically in foot regenerating tissue compared to head regenerating tissue at 12 hpa. FR-specific_24hpa, contains genes up regulated specifically in foot regenerating tissue compared to head regenerating tissue at 24 hpa. FR-specific_48hpa, contains genes up regulated specifically in foot regenerating tissue compared to head regenerating tissue at 48 hpa. HR-specific_3hpa, contains genes up regulated specifically in head regenerating tissue compared to foot regenerating tissue at 3 hpa. HR-specific_12hpa, contains genes up regulated specifically in head regenerating tissue compared to foot regenerating tissue at 12 hpa. HR-specific_24hpa, contains genes up regulated specifically in head regenerating tissue compared to foot regenerating tissue at 24 hpa. HR-specific_48hpa, contains genes up regulated specifically in head regenerating tissue compared to foot regenerating tissue at 48 hpa. Cells with NA values in sheet columns represent Not Available values.

Supplementary Data 5. Excel workbook containing ortholog transcripts between H. oligactisand H. vulgaris that were clustered by the OrthClust pipeline, along with their annotations and expression levels in foot and head regeneration time courses. Each separate sheet contains the transcripts grouped in each cluster from cluster 1 to 9. Cells with NA values in sheet columns represent Not Available values.

Supplementary Data 6. Comma separated values file containing 1,773 genes with differential expression patterns grouped into 9 modules by maSigPro pipeline. Cells with NA values in sheet columns represent Not Available values.

Supplementary Data 7. Excel workbook containing Gene Ontology (GO) term enrichment analysis for all 9 modules in Supplementary_data_6.csv. Each separate sheet contains the enrichment results for each of the 9 modules obtained with the maSigPro pipeline.

Supplementary Data 8 Fasta format file containing all the assembled transcripts used astra nscriptomic reference for H. oligactis used in our study.
