# Supplementary material for: Wnt signaling restores evolutionary loss of robust foot regeneration rates in Hydra
Source: Nat Commun. 2025 Dec 10;16:11447. doi: 10.1038/s41467-025-66299-2 (PMC12748876; doi:10.1038/s41467-025-66299-2)
Supplement: Supplementary file 4 — Supplementary Data 1-8 [file 41467_2025_66299_MOESM4_ESM.zip › Supplementary_Data_Files/README.rtf]

# Description of the files and directories contained in this repository:## Notes: column names and other acronyms in files:hpa: hours post amputationID: granscript name in H. oligactis transcriptomic reference.logFC: base 2 logarithimic fold change from glmTreat test.unshrunk.logFC: base 2 logarithmic fold change without shrinkage applied.logCPM: logaratimic counts per million from edgeR.Pvalue: probability of effect value from glmTreat test.FDR: false discovery rate from adjusted p.value from glmTreat test.Full_name: long gene name from reciprocal blasting results against Swiss prot database.Gene_name: short name from reciprocal blasting results against Swiss prot database.H_sapiens: name of the human ortholog from OrthoFinder pipeline.P_Name: human protein name.G_Name: human gene name.GO_BP: Gene Onthology Biological Process annotated terms for gene.Description: annotated gene function.H_vulgaris105: transcript ID of H. vulgaris ortholog from      Jack F Cazet Adrienne Cho Celina E Juliano (2021) Generic injuries are sufficient to induce ectopic Wnt organizers in Hydra eLife 10:e60562.FR: foot regenerationHR: head regenerationhpg: hours post graftingALP: alsterpaullone-treated aboral injured tissue.N: number of genes in maSigPro ClusterX: number of gnees in Gene Ontology term.Gene_ratio: Gene enrichmente quotient, N/X.P: p-value of enrichment test.logPvalue: logarithmic p-value of enrichment test.P-adj: adjusted p-value for multicomparison test.logQvalue: logarithmic adjusted p-value.attrib ID: ID for the enriched gene ontology terms.attrib name: description for Gene ontology terms.sort.rotation: rotation value for factors in PC1.cluster.mSP: number of cluster from maSigPro analysis.LOD: base 10 logarithm of odds ratio.Supplementary_Data_1.xlsx. Excel workbook containing two lists for foot-specific genes (Foot-specific genes sheet) and head-specific genes (Head-specific genes sheet) in H. oligactis. Cells with NA values in sheet columns represent Not Available values.Supplementary_Data_2.csv. Comma separated values file containing genes loaded onto PC1 from a Principal Component Analysis from Head regeneration RNA-seq samples in H. oligactis. Cells with NA values in sheet columns represent Not Available values.Supplementary_Data_3.csv. Comma separated values file containing genes loaded onto PC1 from Principal Component Analysis from Foot regeneration RNA-seq samples in H. oligactis. Cells with NA values in sheet columns represent Not Available values.Supplementary_data_4.xlsx. Excel workbook containing lists of Differentially Expressed Genes along with their functional annotations from all comparisons listed in the study. Each comparison is contained in a separate sheet: FR-specific_3hpa, contains genes up regulated specifically in foot regenerating tissue compared to head regenerating tissue at 3 hours post amputation (hpa). FR-specific_12hpa, contains genes up regulated specifically in foot regenerating tissue compared to head regenerating tissue at 12 hpa. FR-specific_24hpa, contains genes up regulated specifically in foot regenerating tissue compared to head regenerating tissue at 24 hpa. FR-specific_48hpa, contains genes up regulated specifically in foot regenerating tissue compared to head regenerating tissue at 48 hpa. HR-specific_3hpa, contains genes up regulated specifically in head regenerating tissue compared to foot regenerating tissue at 3 hpa. HR-specific_12hpa, contains genes up regulated specifically in head regenerating tissue compared to foot regenerating tissue at 12 hpa. HR-specific_24hpa, contains genes up regulated specifically in head regenerating tissue compared to foot regenerating tissue at 24 hpa. HR-specific_48hpa, contains genes up regulated specifically in head regenerating tissue compared to foot regenerating tissue at 48 hpa. Cells with NA values in sheet columns represent Not Available values.Supplementary_data_5.xlsx. Excel workbook containing ortholog transcripts between H. oligactis and H. vulgaris that were clustered by the OrthClust pipeline, along with their annotations and expression levels in foot and head regeneration time courses. Each separate sheet contains the transcripts grouped in each cluster from cluster 1 to 9. Cells with NA values in sheet columns represent Not Available values.Supplementary_data_6.csv. Comma separated values file containing 1,773 genes with differential expression patterns grouped into 9 modules by maSigPro pipeline. Cells with NA values in sheet columns represent Not Available values.Supplementary_data_7.xlsx. Excel workbook containing Gene Ontology (GO) term enrichment analysis for all 9 modules in Supplementary_data_6.csv. Each separate sheet contains the enrichment results for each of the 9 modules obtained with the maSigPro pipeline.Supplementary_data_8.fa. Fasta format file containing all the assembled transcripts used as transcriptomic reference for H. oligactis used in our study.
