# Supplementary material for: Wnt signaling restores evolutionary loss of robust foot regeneration rates in Hydra
Source: Nat Commun. 2025 Dec 10;16:11447. doi: 10.1038/s41467-025-66299-2 (PMC12748876; doi:10.1038/s41467-025-66299-2)
Supplement: Supplementary file 5 — Reporting Summary [file 41467_2025_66299_MOESM5_ESM.pdf]

nature portfolio

Corresponding author(s): Celina E. Juliano

Last updated by author(s): Oct 29, 2025

Reporting Summary

Nature Portfolio wishes to improve the reproducibility of the work that we publish. This form provides structure for consistency and transparency in reporting. For further information on Nature Portfolio policies, see our Editorial Policies and the Editorial Policy Checklist.

Statistics

For all statistical analyses, confirm that the following items are present in the figure legend, table legend, main text, or Methods section.

n/a	Confirmed	
☐	☒	The exact sample size (*n*) for each experimental group/condition, given as a discrete number and unit of measurement
☐	☒	A statement on whether measurements were taken from distinct samples or whether the same sample was measured repeatedly
☐	☒	The statistical test(s) used AND whether they are one- or two-sided<br>*Only common tests should be described solely by name; describe more complex techniques in the Methods section.*
☒	☐	A description of all covariates tested
☐	☒	A description of any assumptions or corrections, such as tests of normality and adjustment for multiple comparisons
☐	☒	A full description of the statistical parameters including central tendency (e.g. means) or other basic estimates (e.g. regression coefficient) AND variation (e.g. standard deviation) or associated estimates of uncertainty (e.g. confidence intervals)
☐	☒	For null hypothesis testing, the test statistic (e.g. *F*, *t*, *r*) with confidence intervals, effect sizes, degrees of freedom and *P* value noted<br>*Give P values as exact values whenever suitable.*
☒	☐	For Bayesian analysis, information on the choice of priors and Markov chain Monte Carlo settings
☒	☐	For hierarchical and complex designs, identification of the appropriate level for tests and full reporting of outcomes
☒	☐	Estimates of effect sizes (e.g. Cohen's *d*, Pearson's *r*), indicating how they were calculated

Our web collection on statistics for biologists contains articles on many of the points above.

Software and code

Policy information about availability of computer code

Data collection	Sequencing was obtained using NovaSeq-5000 technology and its proprietary software.
Data analysis	All scripts used in this study are available both as a git repository at https://github.com/cejuliano/oligactis_foot_regeneration. This GitHub release version can be cited using DOI: https://doi.org/10.5281/zenodo.17476370.<br>Published software packages and pipelines were used a across all the analyses. For trimming and mapping RNA-seq reads we used published Linux-based softwares: Trimmomatic v 0.36 and Rsem v1.1.14. For basic differential gene expression analysis we used EdgeR v. 4.0.16, limma v.3.58.1 and sva v.3.50.0 packages for R. For co-expression analysis we used OrthoClust package v.1.0 in R and for gene expression pattern clustering we used maSigPro package v.1.74.0 in R. Confocal images were analyzed with Fiji v.1.54.

For manuscripts utilizing custom algorithms or software that are central to the research but not yet described in published literature, software must be made available to editors and reviewers. We strongly encourage code deposition in a community repository (e.g. GitHub). See the Nature Portfolio guidelines for submitting code & software for further information.

Data

Policy information about availability of data

All manuscripts must include a data availability statement. This statement should provide the following information, where applicable:

- Accession codes, unique identifiers, or web links for publicly available datasets
- A description of any restrictions on data availability
- For clinical datasets or third party data, please ensure that the statement adheres to our policy

FASTQ files of raw RNA-seq reads and raw counts for RNA-seq are available through the Gene Expression Omnibus under the Bio Project accession number: PRJNA1231128.

Research involving human participants, their data, or biological material

Policy information about studies with human participants or human data. See also policy information about sex, gender (identity/presentation), and sexual orientation and race, ethnicity and racism.

Reporting on sex and gender	NA
Reporting on race, ethnicity, or other socially relevant groupings	NA
Population characteristics	NA
Recruitment	NA
Ethics oversight	NA

Note that full information on the approval of the study protocol must also be provided in the manuscript.

Field-specific reporting

Please select the one below that is the best fit for your research. If you are not sure, read the appropriate sections before making your selection.

[x] Life sciences [ ] Behavioural & social sciences [ ] Ecological, evolutionary & environmental sciences

For a reference copy of the document with all sections, see nature.com/documents/nr-reporting-summary-flat.pdf

Life sciences study design

All studies must disclose on these points even when the disclosure is negative.

Sample size	For experiments were statistical analysis was a needed to draw conclusions we use a conservative experimental design based on extensive historical performance of our assays: A minimal N=24 animals per condition in each of three independent biological replicates performed on different days. No sample size calculation for power analysis was performed a priori given that the number of animals is not as much of a limitation in Hydra work compared to work on other animal species. We selected number sizes based on previous reports in Hydra (referenced in the main text) where smaller number of animals were sufficient to detect the effects of different treatments on regeneration and secondary axis formation (15,17,19,20,35).This approach prioritizes independence and reproducibility, captures between-batch variation that dominates the error structure in Hydra work, and avoids underpowered studies without expending e,ffort on uncertain model assumptions. We report exact n for every experiment, include all data points, use effect sizes with confidence intervals, and rely on nonparametric or mixed-effects analyses as appropriate. Given these features of the system and our standardized large-N design, a separate prospective power calculation adds little value to the rigor or interpretability of our results.
Data exclusions	No data were excluded from the analysis.
Replication	Sequencing experiments were performed with at least three biological replicates. Bisections and other phenotypic assays were performed using at least three batches. RNA in situ hybridization experiments were performed twice with 20 animals per probe per condition. Experiments on each batch were performed at different times by the same experimenter. In all cases were different batches of biological replicates were tested our results where replicated successfully.
Randomization	Adult animals used for experimenting were selected from large cultures. Further, after selection animals were allocated at random in different biological replicates. In the case of regeneration experiments animals under different conditions were allocated into different treatment groups at random and then put into different wells in 24 well-plates by assigning random positions using R.
Blinding	Blinding of experimenters was not used since the aims of this work do not involve outcomes subject to individual appreciation. All measurements were highly quantitative and performed equally for all batches withing experimental groups. In addition, computational methods were applied to samples using the same parameters regardless of sample groups, decreasing the need for blinding.

Reporting for specific materials, systems and methods

We require information from authors about some types of materials, experimental systems and methods used in many studies. Here, indicate whether each material, system or method listed is relevant to your study. If you are not sure if a list item applies to your research, read the appropriate section before selecting a response.

Materials & experimental systems

n/a	Involved in the study	
x		Antibodies
x		Eukaryotic cell lines
x		Palaeontology and archaeology
	x	Animals and other organisms
x		Clinical data
x		Dual use research of concern
x		Plants

Methods

n/a	Involved in the study	
x		ChIP-seq
x		Flow cytometry
x		MRI-based neuroimaging

Animals and other research organisms

Policy information about studies involving animals; ARRIVE guidelines recommended for reporting animal research, and Sex and Gender in Research

Laboratory animals	Hydra oligactis (strain: Innsbruck 12) collected from Innsbruck, Austria. and adapted to aboratory culture by Bert Hobmayer, previous to donation. Hydra vulgaris (strain: AEP) has been kept in laboratories and reported extensively in other works (19). H. oxycnida (strain: AUT08a, Vienna, Austria), H. hymanae (strain: CA25a, Eel River, California, USA) and H. viridissima (strain: 665a, Peru) were all collected and provided by Ruthie B. Spencer and Daniel E. Martínez and Robert E. Steele.
Wild animals	NA
Reporting on sex	Hydra cultures contain both female and male animals, which cannot be indentified unless sexual gonad production is induced, which in turns is counterproductive for the purpose of observing regeneration, which happens only in the asexual stage.
Field-collected samples	NA
Ethics oversight	Ethical approval was not require to work with the invertebrate animals used in this study.

Note that full information on the approval of the study protocol must also be provided in the manuscript.

Plants

Seed stocks	NA
Novel plant genotypes	NA
Authentication	NA
