# Supplementary material for: Wnt signaling restores evolutionary loss of robust foot regeneration rates in Hydra
Source: Nat Commun. 2025 Dec 10;16:11447. doi: 10.1038/s41467-025-66299-2 (PMC12748876; doi:10.1038/s41467-025-66299-2)
Supplement: Supplementary file 6 — Source data [file 41467_2025_66299_MOESM6_ESM.zip › Source_Data_ALL/README.rtf]

# Description of the files and directories contained in this repository:## Notes: column names and other acronyms in files:hpa: hours post amputationID: granscript name in H. oligactis transcriptomic reference.logFC: base 2 logarithimic fold change from glmTreat test.unshrunk.logFC: base 2 logarithmic fold change without shrinkage applied.logCPM: logaratimic counts per million from edgeR.Pvalue: probability of effect value from glmTreat test.FDR: false discovery rate from adjusted p.value from glmTreat test.Full_name: long gene name from reciprocal blasting results against Swiss prot database.Gene_name: short name from reciprocal blasting results against Swiss prot database.H_sapiens: name of the human ortholog from OrthoFinder pipeline.P_Name: human protein name.G_Name: human gene name.GO_BP: Gene Onthology Biological Process annotated terms for gene.Description: annotated gene function.H_vulgaris105: transcript ID of H. vulgaris ortholog from      Jack F Cazet Adrienne Cho Celina E Juliano (2021) Generic injuries are sufficient to induce ectopic Wnt organizers in Hydra eLife 10:e60562.FR: foot regenerationHR: head regenerationhpg: hours post graftingALP: alsterpaullone-treated aboral injured tissue.N: number of genes in maSigPro ClusterX: number of gnees in Gene Ontology term.Gene_ratio: Gene enrichmente quotient, N/X.P: p-value of enrichment test.logPvalue: logarithmic p-value of enrichment test.P-adj: adjusted p-value for multicomparison test.logQvalue: logarithmic adjusted p-value.attrib ID: ID for the enriched gene ontology terms.attrib name: description for Gene ontology terms.sort.rotation: rotation value for factors in PC1.cluster.mSP: number of cluster from maSigPro analysis.LOD: base 10 logarithm of odds ratio.Source data file 1. Text file containing the accession numbers and sequences for CO1 used to build the phylogenetic tree in Figure 1a.Source data file 2. Excel workbook containing head and foot regeneration data for the experiments shown in Figure 1 g-k.Source data file 3. Excel workbook containing differentially expressed gene tables for the conditions compared in Figure 2 c-d.Source data file 4. Comma separated values file containing normalized log2 CPM used to plot Figure 2 e-f.Source data file 5. Excel workbook containing log2 Fold Change values from comparisons in Figure 2 g-j.Source data file 6. Excel workbook containing log2 CPM for selected foot-specific genes in H. vulgaris and H. oligactis shown in Figure 2 k-l.Source data file 7. Comma separated values file containing FPKM for H. oligactis transcripts used for Orthoclust analysis.Source data file 8. Comma separated values file containing FPKM for H. vulgaris transcripts used for Orthoclust analysis.Source data file 9. Comma separated values file containing CPM for H. vulgaris transcripts for gene expression pattern comparisons.Source data file 10. Excel workbook containing secondary axis formation frequency from grafting in H. vulgaris and H. oligactis Figure 3M. Data for each timepoint and species are given in separate sheets.Source data file 11. Excel workbook containing foot and head regeneration percentages found in H. vulgaris after 12 hours of iCRT14 treatment or DMSO as shown in Figure 4 b-c.Source data file 12. Comma separated values file containing foot regeneration percentages for H. oligactis treated with the different ALP concentrations shown in Figure 4B.Source data file 13. Comma separated values file containing normalized log2CPM used to plot data shown in Figure 5 B.Source data file 14. Excel workbook containing fold change gene tables for the timepoints comparing foot regeneration in DMSO and ALP in Figure 5C and D.Source data file 15. Excel workbook containing Gene Ontology enriched terms for maSigPro modules shown in Figure 5F and G.Source data file 16. Comma separated values file containing log fold change values for the expression of TFs shown in Figure 5H at the timepoints indicated.Source data file 17. Excel workbook containing gene expression data from RNA-seq analysis for the 154 foot-specific gene orthologs and 63 randomly-selected orthologs in H. vulgaris and H. oligactis.Source Data file 18. Excel workbook containing head and foot regeneration data for H. oligactis treated with ALP 5 µM or DMSO.Source Data file 19. Comma separated values file containing raw foot regeneration data for H. oligactis treated with iCRT14 or DMSO using different treatment schemes.
